# Foraging in the Darkness of the Southern Ocean: Influence of Bioluminescence on a Deep Diving Predator

**DOI:** 10.1371/journal.pone.0043565

**Published:** 2012-08-29

**Authors:** Jade Vacquié-Garcia, François Royer, Anne-Cécile Dragon, Morgane Viviant, Frédéric Bailleul, Christophe Guinet

**Affiliations:** 1 Centre d’Etudes Biologiques de Chizé (CEBC), CNRS UPR 1934, Villiers-en-bois, France; 2 Collecte Localisation Satellites (CLS), Ramonville Saint-Agne, France; Institut Pluridisciplinaire Hubert Curien, France

## Abstract

How non-echolocating deep diving marine predators locate their prey while foraging remains mostly unknown. Female southern elephant seals (SES) (*Mirounga leonina*) have vision adapted to low intensity light with a peak sensitivity at 485 nm. This matches the wavelength of bioluminescence produced by a large range of marine organisms including myctophid fish, SES’s main prey. In this study, we investigated whether bioluminescence provides an accurate estimate of prey occurrence for SES. To do so, four SES were satellite-tracked during their post-breeding foraging trip and were equipped with Time-Depth-Recorders that also recorded light levels every two seconds. A total of 3386 dives were processed through a light-treatment model that detected light events higher than ambient level, i.e. bioluminescence events. The number of bioluminescence events was related to an index of foraging intensity for SES dives deep enough to avoid the influence of natural ambient light. The occurrence of bioluminescence was found to be negatively related to depth both at night and day. Foraging intensity was also positively related to bioluminescence both during day and night. This result suggests that bioluminescence likely provides SES with valuable indications of prey occurrence and might be a key element in predator-prey interactions in deep-dark marine environments.

## Introduction

Understanding the dynamic relationship between prey and predators is a key topic in ecology [1]. In many natural cases, prey are clustered in patches [2] with no clear boundaries. These patches can only be defined as areas where the local resource density is higher than the mean overall resource density [3], [4]. In these continuous, yet patchy environments, such as marine environments for instance, efficient predators are likely to focus their search effort to areas of high density of prey [4]–[6].

Numerous studies have investigated the foraging behaviour of marine predators (such as birds and mammals) in relation to physical (e.g. temperature) and biological (e.g. phytoplankton) parameters used as indirect indicators of prey distribution [7]–[9]. However, only few studies looked at the relationships between foraging behaviour of diving predators and direct indices of the presence of prey (e.g. videos) [10], [11].

Marine predators can cue in on signals emitted by prey to estimate the local densities or quality of prey. They rely on their sensory systems such as olfaction [12], [13], echolocation [14] or, in most of the cases, vision [15], [16] to detect these signals. Unlike odontocetes, pinnipeds do not echolocate to find their prey [17]. This raises the question of how deep-diving pinnipeds manage to locate their prey in the deep dark ocean. Previous studies have shown that some pinnipeds that feed at night or during deep dives, such as elephant seals, have a vision adapted to low light intensity characterized by a sensitivity peak at 485 nm ( =  λ max). This corresponds to the blue light produced by bioluminescent marine organisms and could suggest a vision-based predation [18]–[20].

Southern elephant seals (*Mirounga leonina*) (SES hereafter) are the largest of pinnipeds. They spend around 10 months at sea and come back ashore only to breed in October, or to molt in January. When foraging at sea, SESs dive continuously, sometimes deeper than 1500**m [21]. They forage over broad distances [21], [22].

Both Fatty Acid and stable isotopes analyses suggest that myctophids (or lantern fish) are the main prey of both adult females [23] and juvenile males [24]. Stomach content analyses of female elephant seals from King George Island and Heard Island [25], [26] also reveal that the most common myctophids species in their diet were *Gymnoscopelus nicholsi, Electrona Antarctica and E. calsbergi.* These 3 species also happen to be the 3-most abundant myctophids within the Southern Ocean [27].

Myctophids have a large number of photophores (light-producing organs) located in ventrolateral rows of their body and head, which gives them the capacity to be bioluminescent. Bioluminescence emitted by myctophids can be of two sorts and serve different purposes: bright and fast flashes are used for communication (i.e. intra-specific and sex identification) or to startle potential predators, while glow emissions are used to mask the fish silhouette from predators underneath (counter-illumination) [28]–[30]. Previous work showed that SESs have large eyes with a capacity for a wide range of pupillary dilation [19] and rapid adjustments to the darkness [20]. Their retina contains a deep-sea rhodopsin with a maximum sensitivity (ability to react to a stimulus) at 485 nm [18] matching the λmax of the visual pigments of their myctophid prey (*Gymoscopelus nicholsi*: λmax = 489 nm, *Electrona antarctica*: λmax = 488 nm, *E. carlsbergi*: λmax = 485 nm) [31].

The objective of this study was to assess whether the number of bioluminescence events detected during deep dives provides an accurate qualitative index of the visited foraging areas. We focused on the Kerguelen SES population. We investigated the foraging activity of females SES at depth in relation to the amount of *in situ* bioluminescence detected during a dive by a highly sensitive light sensor. Due to limited sample size and to avoid confounding factors such as the effects of foraging habitat features (i.e two third of SES females foraging in Polar frontal zone vs one third in Antarctic waters) [24], [32], the analyses were restricted to female SES foraging within the polar frontal zone.

**Figure 1:**
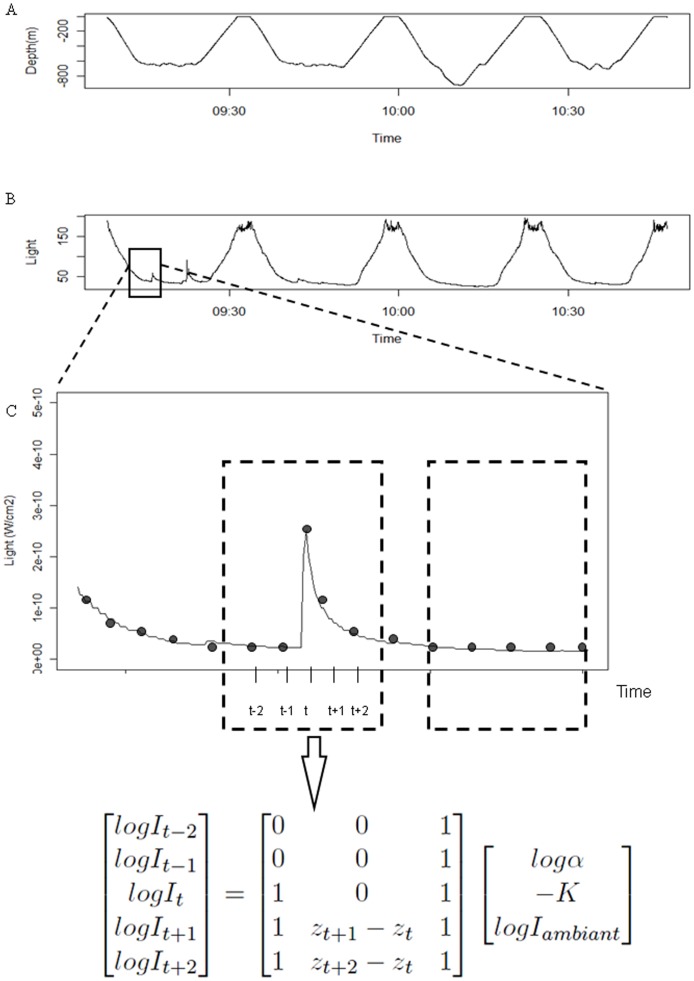
Detection of bioluminescence events. A. An individual time-depth profile zoomed on four consecutive dives. B. Light values associated with dives. C. The method used to detect bioluminescence events. Each point is associated to a light value (I), a depth value (Z) and a time (t). A restricted window (dashed lines) was moved point by point along the light profile. Within a window, the central point corresponded to the I(t) light value. I_ambient_ corresponded to the expected light level when there is no bioluminescence event and was calculated using the two first light values within the window. α(t) corresponded to the ratio between the I_ambient_ and the I(t) light values. The Kt corresponded to the coefficient associated with the decrease of the light following an event.

**Figure 2:**
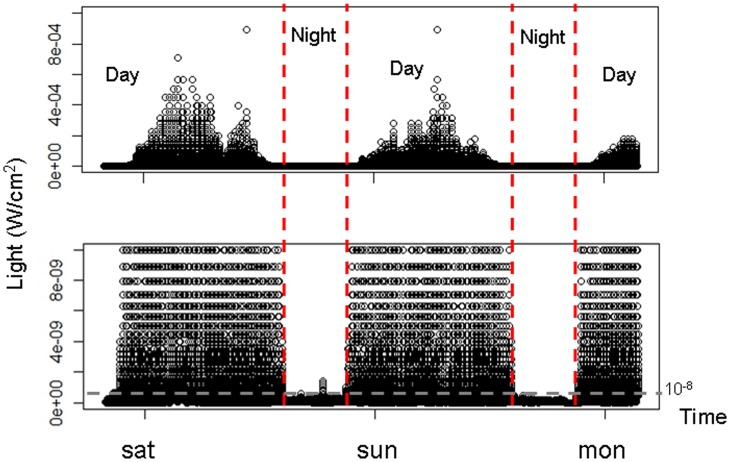
Separating of day and night periods. These graphs show light profile during 2 complete days and the beginning to a third. The dotted grey line represents the day and night separation threshold. For a dive, if the surface light is higher than this threshold (10^−8 ^W.cm^−2^), the dive is considered as a day dive and if the surface light is lower than this threshold, the dive is considered as night dive. The dotted red vertical lines represent the separation between day and night period.

## Methods

### Ethics Statement

Our study on elephant seals was approved and authorized by the ethics committee of the French Polar Institute (Institut Paul Emile Victor – IPEV) in May 2008. This Institute does not provide any permit number or approval ID, however animals were handled and cared for in total accordance with the guidelines and recommendations of this committee (dirpol@ipev.fr).

**Figure 3:**
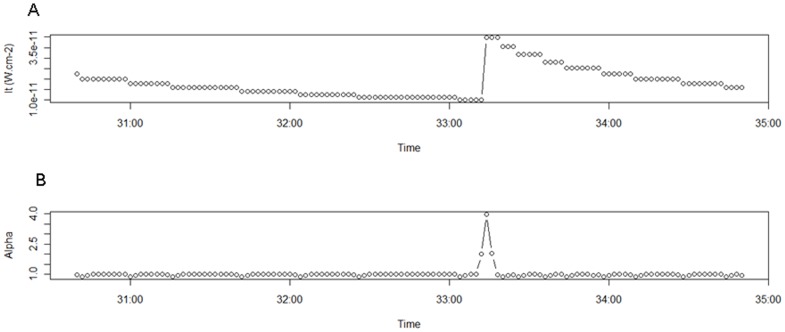
Light profile of a bioluminescence events and the α(t) profile. A. The light profile of one bioluminescence event. B. The α(t) profile of this bioluminescence event. α(t) is greater 1 during the increasing light phase of a bioluminescence event.

### Deployment of Devices and Data Collected

The foraging ecology of Southern elephant seals in Kerguelen has been investigated since 2003 when annual deployments of CTD-SRLDs started (Argos-linked conductivity–temperature–depth-Satellite Relayed Data Logger/Sea Mammal Research Unit –SMRU-, University of St Andrews Scotland). In 2009 however, we deployed new devices that also included a fluorescence sensor, combined with MK9 -Time Depth Recorders (Wildlife Computers, Washington, USA). We thus recorded fine scale diving behaviors of five SES females in addition to high resolution measures (every 2 sec) of temperature and light during their entire post-breeding foraging trip.

**Figure 4:**
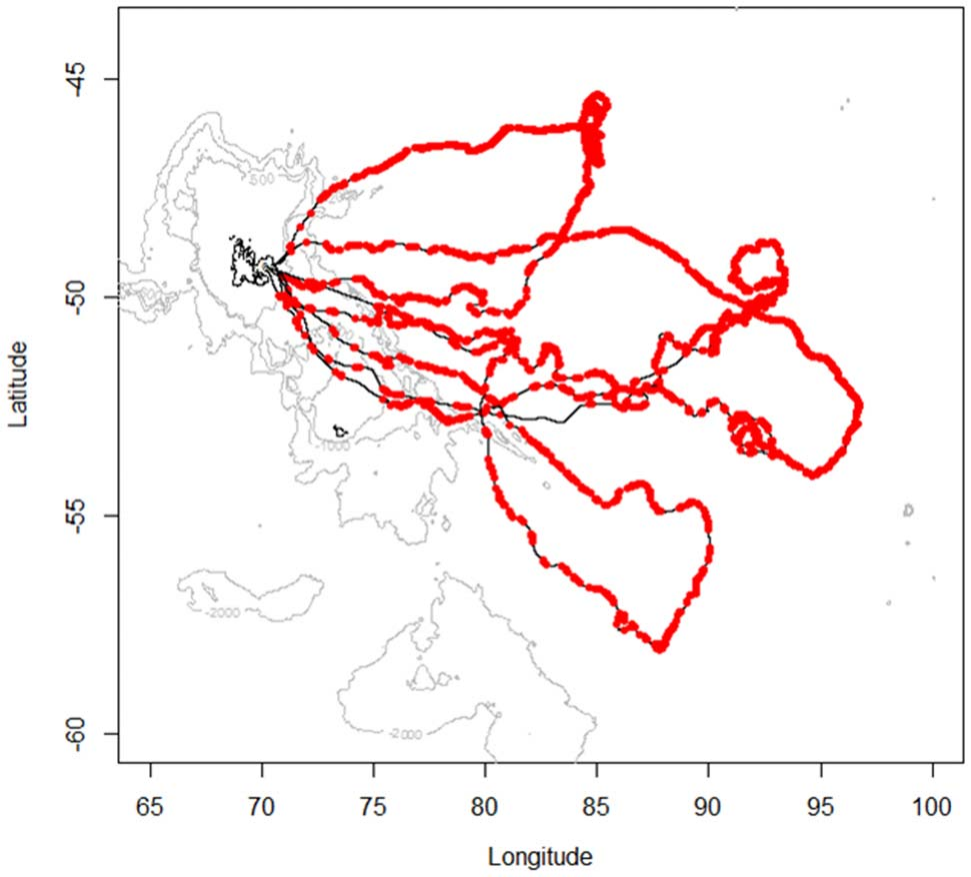
Post-breeding foraging trips of 4 female southern elephant seals. These females were equipped in October 2009 (solid black lines). Isobaths of 500 m, 1000 m and 2000 m deep are illustrated in light grey. Kerguelen island contour is depicted in black line. Red dots correspond to points into dives including at least one significant bioluminescence event (s).

The light sensor (Hamamatsu S2387 series photodiode) was used with a 470–590 nm filter. This light sensor was able to measure changes in light under very low light conditions: its detection capacity ranged from 10 to 250 in raw values, corresponding to a range of 10^−11^–10^−1^ W.cm^−2^.

**Figure 5:**
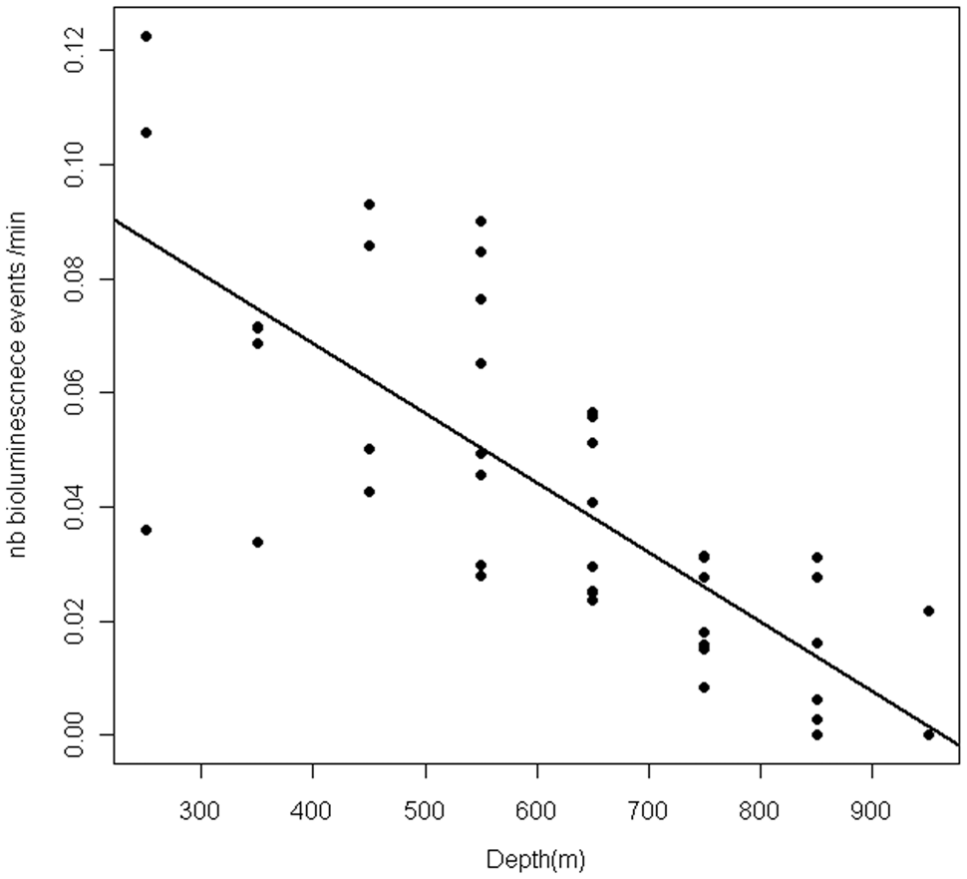
Bioluminescence into the water mass. The graph represents the distribution of the bioluminescence events according to the depth. The number of events was corrected by the time spent by all the individuals in each depth category. The black line shows the linear regression between the number of bioluminescence events per minute and depth.

Animals were captured with a canvas head-bag and anesthetized using a 1∶1 combination of Tiletamine and Zolazepam (Zoletil 100) injected intravenously [33], [34]. MK9 were first attached to the CTDs, and then both CTDs and MK9 tags were glued on the seal’s head using quick-setting epoxy (Araldite AW 2101). CTDs were oriented towards the head of the animal, while MK9 were oriented backwards so that light sensors were turned towards the backside of the animals.

**Figure 6:**
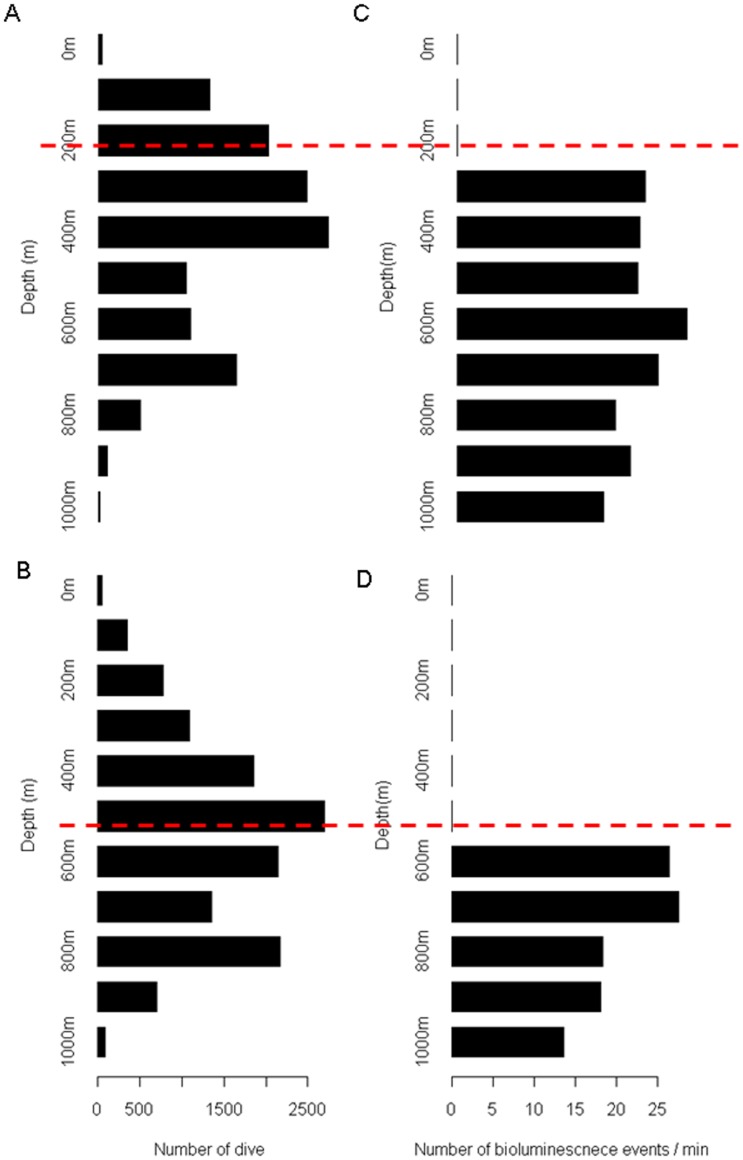
Dive depth and the depth of bioluminescence events. A–B. Histogram of the dives depth at night (A) and during the day (B). C–D. Histogram of the depth of bioluminescence event per minute at night (C) and during the day (D). Events were only detected at night below a threshold of 250 m and during the day below a threshold of 500 m (shown by a dashed red line).

**Figure 7:**
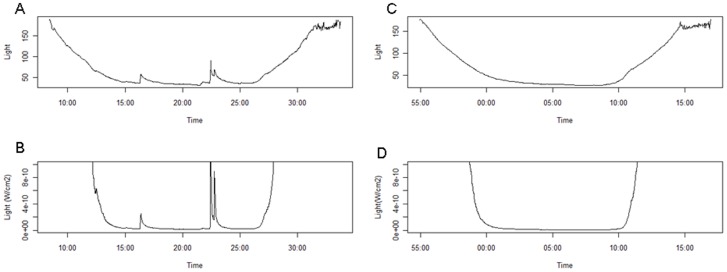
Bioluminescence and foraging intensity. A–B. A light profile (raw values = A; W/cm^2^ = B) for a dive with many bioluminescence events and a high foraging index, controlling for depth and bottom time. C–D. A light profile (raw values = C; W/cm^2^ = D) for a dive without bioluminescence events and a low foraging index, controlling for depth and bottom time.

**Table 1:** Bioluminescence and index of foraging intensity.

	Night		Day	
*Parameters*	*Estimate*	*P-value*	*Estimate*	*P-value*
Bottom Time	1,10E-03	<0.001	−8,32E-05	0.016
Depth	2,23E-03	<0.001	–	
Nb of bioluminescence event	4,81E-02	<0.001	1,10E-01	<0.001
Bottom Time:Depth	−1,70E-06	<0.001	–	
Bottom Time : Nb of bioluminescence event	–		−1,31E-04	<0.001
Depth : Nb of bioluminescence event	–		–	

SES from Kerguelen that feed within the Polar Frontal Zone can forage within two distinct oceanographic domains: 1) the pelagic zone (i.e; deeper than 1000 meter) encompassed between the subantartic and the Polar Front, and 2) the benthic zone over the continental plateau, where diving depths are restricted by the local bathymetry (i.e. shallower than 1000 m). In this study, we only focused on animals that foraged within the pelagic domain.

### Diving Behaviour and Characterization of Foraging

We considered seals diving only for depths of 15 m or more, otherwise they were at the surface. This threshold was chosen to avoid wrongly selecting dives due to subsurface movements of animals. Dives were then divided into three distinct phases using a vertical speed criterion. Descent and ascent phases were defined as movements with a vertical speed greater than 0.4 m.s^−1^ from or toward the surface. The bottom phase was defined as a period between the descent and the ascent phases with a vertical speed lower than 0.4 m.s^−1^ [35], during which, SES could travel upward, downward or horizontally.

**Figure 8:**
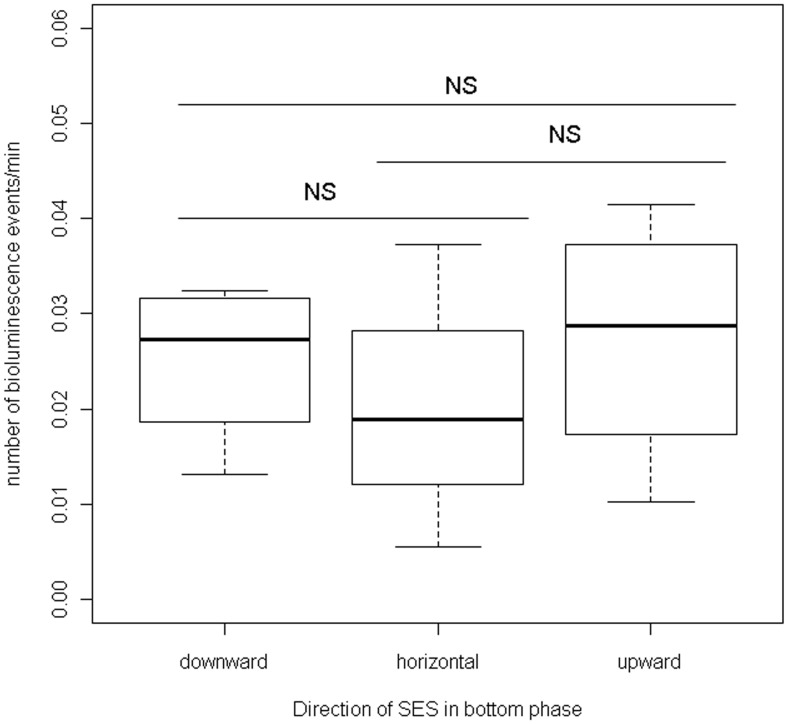
Bioluminescence and predator movement into the bottom phase. This graph represents the number of bioluminescence events encountered in the bottom phase of the dive in a downward, horizontal or upward movement.

We used two factors to define the foraging intensity of each dive [35]: the ascent rate of a dive and the descent rate of the following one. These two factors are known to influence foraging success in a large number of marine predators [36]–[39], (C.Guinet, unpublished data on SES). We combined these variables using a Principal Component Analysis (PCA) to obtain an unique index to the foraging intensity. Principal component 1 (The first axis) explained 78.9% of the total variance and was used as an index of foraging intensity per dive in our analyses.

### Detection of Bioluminescence Events

Typically, light levels measured with a sensor show a typical nycthemeral cycle with variations driven by the sun or the moon. In an oceanic environment, the influence of the sun or the moon on recorded light values is strongly dependent on depth (Figure 1A and B). The environment light (i.e. ambient light) reaches nearly constant low values at depths of 550 m during the day and 250 m at night. Therefore, any sudden increase in the ambient light level at depths deeper than these limits corresponds to the occurrence of a bioluminescent event around the SES. We consequently kept bioluminescent events only at depths deeper than the ones aforementioned for analysis. To fit the international metric system, the raw values measured by the sensors were converted into W.cm^−2^ using the following equation provided by the manufacturer (Wildlife Computers): 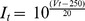 where Vt  =  light value from the sensor and It  =  light value converted in W.cm^−2^. Night periods were defined by surface light levels lower than 10^−8^ W.cm^−2^ and day periods by a surface light higher than 10^−8^ W.cm^−2^ (Figure 2).

Bioluminescent events were characterized by an abrupt increase followed by a progressive decrease in light levels (Figure 1C). Bioluminescence events were mainly detected and characterized from the abrupt increase phase, rather than from the entire signal (increase and decrease phases). However, the decrease phase was still useful for parameter calculations (see below). A system of running windows over 10 sec (5 data points) with an increment of 2 s (i.e. 1 data point) was used to determine the beginning and the end of bioluminescence events, the end being here the point of highest light intensity (the decreasing phase is omitted) (Figure 1C). The mid-point of the 5-point running window was named time t, with the previous two times t-1 and t-2 and the following two t+1 and t+2respectively. Light data were log transformed for analysis to buffer the wide range of recorded values (10^−11^ to 10^−1^ W.cm^−2^).Three parameters characterized the central point t:

The ambient light, I _ambient_
A fast increase in light, α_t_
A slower decrease in light, K.

These 3 parameters were estimated using the qr.solve function of the package base in R as follows:
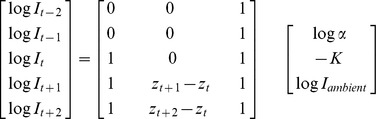
with I_t_ and I_ambient_ in W.cm^−2^, K in m^−1^ and Z the depth at time t in m. (Figure 1.)

α_t_ can be estimated from the matrix as follows:
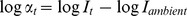





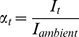
.

Bioluminescence events started when α_t_ was higher than 1 and lasted as long as it remained above this threshold (Figure 3). Once bioluminescence events were detected, we selected only the significant ones, i.e.events for which the ratio between maximum light into the event and ambient light at the beginning point of the event was higher than 1.26 (sensor resolution). The others were discarded as sensor noise and not included in the analysis. Event intensity was then calculated as the difference between the maximum light and ambient light at the beginning point of the event.

### Statistical Analysis

#### Bioluminescence within the water column

The water column was divided into 100 m-deep layers: six of them at night (from 250–350 to 850–950 m) and 4 of them for the daylight period (from 550–650 to 950–1050 m every 100 m). The relationship between the total number of bioluminescence events per unit of time spent within a depth layer, the depth layer, and the time of the day (day or night) was determined with linear regressions (‘stat’ package in R.2.10.1).

#### Bioluminescence and foraging intensity

As foraging is usually related to the bottom phase of a dive [40]–[44], only bioluminescence events at the bottom phase of dives were kept in the analyses. The bottom time and the depth of a dive are known to be linked to foraging activity [35]. Both variables were found here to be correlated to the number of bioluminescence events met during dives. In order to account for this, the index of foraging intensity of dives was investigated in relation to the bottom time, the depth and the number of bioluminescence events of dives using generalized linear mixed models at night and day separately (nlme package in R 2.10.1) [45]. Individuals were included as random factors and we accounted for the temporal correlation in our data using an autoregressive variance-covariance matrix (corAR1). The complete GLMMs were built as such:

*Indexes of foraging intensity ∼ Bottom time + Depth + Number of bioluminescence event + Bottom Time:Depth + Bottom Time:Number of bioluminescence event + Depth: Number of bioluminescence event, random =  ∼ 1|ID, correlation  =  corAR1 ()*.

The best models for night and day were selected using stepwise likelihood ratio tests [46].

#### Bioluminescence and predator movements into the bottom phase

We also investigated the relationship between SES directional movements and bioluminescence. To determine whether bioluminescence events were more likely to be encountered in one direction than another, we analyzed the links between the number of bioluminescence events encountered by SES (and corrected by time), and the direction they followed (i. e. upward, downward and horizontal) using a Wilcoxon test.

## Results

Figure 4 shows the horizontal tracks of the four studied southern elephant seals. These 4 individuals spent on average 65±1 days in oceanic waters for a total foraging trip duration of 75±11 days. Each seal performed on average 71±3 dives per day and 5078±400 dives over the entire foraging trip, 4593±1033 of which in pelagic waters. Bioluminescence events were detectable all along their tracks in the horizontal dimension (Figure 4) and in the vertical dimension (Figure 1A). According to the PCA analysis, indexes of high foraging intensity were characterised by a high ascent rate of the dive and a high descent rate of the following one. On the other hand, indexes of low foraging intensity were characterized by low descent and ascent rates.

### Bioluminescence within the Water Column

A mean of 1234±407 (n = 4) bioluminescence events were detected in the pelagic phase of a foraging trip. The number of detected bioluminescence events decreased with depth similarly both during day and night (Estimate −0.0003479, t-value =  −2.964, p-value = 0.00480). We detected an outlier data point exercising a large leverage effect at night time, however the relationship remained significant after removing this point (Figure 5) (Estimate −1.218e-04; t-value = −8.653 p-value<0.001).

### Bioluminescence and Foraging Intensity

Animals generally dove up to 400 m deep at night and up to 800 m deep during the day. These values corresponded to depths where most of the bioluminescence events were located (Figure 6). On average, each seal performed 4593±1033 dives (n = 4) in pelagic water. Among all pelagic dives performed, a mean 2256±548 dives exceeded the depth threshold set for the detection of bioluminescent events and among them bioluminescence events were detected in 847±246 dives. Therefore a total of 3386 dives were analyzed. The best models in the night and day period showed that the foraging intensity of dives was positively influenced by the number of bioluminescence events met in dives (Figure 7 and Table 1).

### Bioluminescence and Predator Movement into the Bottom Phase

Number of bioluminescence events corrected by time were similar in the downward, horizontal and upward directions (W = 10, p-value = 0.69; W = 11, p-value = 0.49, and W = 6, p-value = 0.69 for comparisons between downward vs horizontal; upward vs horizontal, and upward vs downward respectively) (Figure 8).

## Discussion

Bioluminescence emitted by organisms is a widely-spread phenomenon in marine environment. Our study is one of the first to investigate the relationship between a deep-diving predator and some bioluminescent organisms (in a 470–590 nm range) [47]. Although we do not provide any direct evidence of bioluminescence events, the range of light recorded (470–590 nm, i.e. mainly blue light) included in the marine bioluminescence spectrum (400 to 720 nm). In addition, the depths selected for analyses (i.e. while SES were deep diving and when bioluminescence events could not be detected due to surface light) confirm that the light pulses were most likely bioluminescence events [48]–[50].

This study revealed that bioluminescence in a 470–590 nm range is abundant throughout the polar frontal zone. Numerous taxa such as jelly fishes, crustaceans, squids and fishes can emit bioluminescence in the measured range of wavelength [30], [51] which encompassed SES prey as Mycthophids as well as all the lower trophic levels than myctophids such as krill and dinoflagellates species. Even though we could not relate bioluminescence events to specific SES prey for certain, most bioluminescence event intensities recorded ranged from 0.15 10^−11^ to 0.11 10^−6^ W.cm^−2^. As this window encompasses the emission patterns of the main prey of elephant seals, the Myctophids, (i.e. Myctophids photophores: 10^−7^ W.cm^2)^, [30], it is reasonable to assume that at least one part of bioluminescence events detected were emitted by these prey species. It is even more likely given the large myctophid biomass found in the Southern Ocean south of the 40thS parallel (from 70 to 200 million t) [52]–[54].

Our results show that there is a relationship between this bioluminescence and SES foraging activity as foraging intensity index of seals was positively related to the number of bioluminescence events detected in dive, for both day and night. This suggests that bioluminescence could provide qualitative information of the visited area even though further work is needed to relate bioluminescence to specific oceanographic features. Considering the large possible source of bioluminescence, its occurrence should not be considered as an absolute indicator of SES prey but rather as an index of biological richness of the habitat used by SES. Previous studies have shown, for instance, that species from lower trophic levels have the ability, by producing bioluminescence, to make their predators vulnerable to the attack from higher order predators [30]. Therefore, we argue that SES might also use bioluminescence produced by the lower trophic levels of its own prey (which can be bioluminescent or not) to assess foraging quality of an area.

It is important to note that, seals were also found to exhibit high index of foraging activity in absence of bioluminescence events. It is coherent with the fact that the diet of elephant seals is also composed of non-bioluminescent species [55]–[57]. Thus, while this study focused on the possible role of visual-clues on the foraging behavior of SES females, it is obvious that other sensory systems likely play an important role in prey location. For instance, captive experiments on phocid seals have shown that these animals also use their vibrissaes to detect and locate the vibratory fields produced by fish swimming movements [58]. Highly-developed auditory capacities of elephant seals may also play an important role in locating their prey [59], [60].

Some bioluminescence events may be directly related to the foraging activity of SES, since such events are known to represent a response to an approaching predator such as elephant seals (a light defense) [30]. To avoid predators, some animals use deception or confusion. One deep-sea shrimp is known to spit out bioluminescent slurry to distract attackers [30]. Vampire squids release cloud of bioluminescence to confuse or repel a predator while escaping [61]. Flashlight fish also use the bioluminescent patches under their eyes to help them flee from predators by flashing them off and on while swimming in different directions [30], [62]. Future studies should investigate in greater detail if elephant seals change their tri-dimensional movement patterns in response to these bioluminescence events (i.e. a prey occurrence cues) or prior to the occurrence of these events (i.e. events as a response to an approaching predator).

Our results showed that the number of bioluminescence events decreased with depth both during the day and the night. This trend could be explained by the natural common decrease of biomass with depth [63]. However, contrary to what was expected with the main prey of elephant seals, such as myctophids that display a daily vertical migration pattern, no significant nycthemeral migration was observed. This can be due to the large number of bioluminescent species with different daily ecology and behaviors potentially detected. Our results did not show that seals affected their directionality (downward, horizontal or upward) when approaching a bioluminescence event. This suggests that SES do not use a preferential strategy when faced with bioluminescence, which is consistent with the fact that bioluminescence events can be easily detected from every angle in the total darkness at depth.

It is important to note, however, that light sensors were turned towards the back side of the seals, its detection angle were limited, and measured light only every 2 seconds on a wavelength range limited (470–590 nm). We also only detected bright and fast flashes (related to communication or anti-predatory reaction) rather than glows (counter–illumination behavior). This means that not all bioluminescence events visible to seals were detected and used in our study.

Nevertheless, this study provides important information on the horizontal and vertical distribution of bioluminescent organisms in a 470–590 nm range within the polar frontal zone of the Southern Ocean. It also brings new knowledge and an important baseline regarding the predator-prey interactions through visual cues at depth in the marine environment. While this study it provides essential information, further investigations would be needed to quantitatively relate foraging behavior of elephant seals to bioluminescence. To do so, light sensors with a narrower range of wavelength detection should be paired with accelerometers. This would allow detecting attempt of prey capture at fine scale [64], [65] as well as fine scale SES movement.
